# Glycan biomarkers for Alzheimer disease correlate with T‐tau and P‐tau in cerebrospinal fluid in subjective cognitive impairment

**DOI:** 10.1111/febs.15197

**Published:** 2020-01-14

**Authors:** Sophia Schedin‐Weiss, Stefan Gaunitz, Ping Sui, Qiushi Chen, Stuart M. Haslam, Kaj Blennow, Bengt Winblad, Anne Dell, Lars O. Tjernberg

**Affiliations:** ^1^ Division of Neurogeriatrics Center for Alzheimer Research Department of Neurobiology, Care Sciences, and Society Karolinska Institutet Solna Sweden; ^2^ Department of Life Sciences Imperial College London UK; ^3^ Department of Psychiatry and Neurochemistry the Sahlgrenska Academy at the University of Gothenburg Mölndal Sweden; ^4^ Clinical Neurochemistry Laboratory Sahlgrenska University Hospital Mölndal Sweden; ^5^ Present address: Division of Glycoscience/Department of Chemistry School of Engineering Sciences in Chemistry, Biotechnology and Health KTH Royal Institute of Technology Stockholm Sweden

**Keywords:** Alzheimer disease, biomarkers, cerebrospinal fluid, glycomics, protein glycosylation, tau

## Abstract

Alzheimer disease (AD) is a devastating disease and a global health problem, and current treatments are only symptomatic. A wealth of clinical studies support that the disease starts to develop decades before the first symptoms appear, emphasizing the importance of studying early changes for improving early diagnosis and guiding toward novel treatment strategies. Protein glycosylation is altered in AD but it remains to be clarified why these alterations occur and how they affect the disease development. Here, we used a glycomics approach to search for alterations in protein glycosylation in cerebrospinal fluid (CSF) in AD compared with nondemented controls. Using both matrix‐assisted laser desorption ionization‐time of flight and liquid chromatography–electrospray mass spectrometry, we observed an increase in N‐glycans carrying bisecting N‐acetylglucosamine in AD. Based on those findings, we designed an enzyme‐linked multiwell plate assay to quantify N‐glycans binding to the lectin *Phaseolus vulgaris* Erythroagglutinin (PHA‐E), which is specific for N‐glycans containing bisecting N‐acetylglucosamine. Using this assay, we found a similar increase in CSF in AD compared with controls. Further analysis of CSF from 242 patients with subjective cognitive impairment (SCI), mild cognitive impairment (MCI), or AD dementia revealed significantly increased binding to PHA‐E in MCI and AD compared to SCI. Interestingly, PHA‐E binding correlated with CSF levels of phosphorylated tau and total tau and this correlation was most prominent in the SCI group (*R* = 0.53–0.54). This study supports a link between N‐glycosylation, neurodegeneration, and tau pathology in AD and suggests that glycan biomarkers have potential to identify SCI cases at risk of developing AD.

## Graphical abstract

Glycomics analyses showed alterations in the N‐glycan pattern in cerebrospinal fluid (CSF) in Alzheimer disease as compared to controls. The levels of several N‐linked glycans carrying bisecting N‐acetylglucosamine were increased in AD. A multiwell plate enzyme‐linked lectin assay denoted enzyme‐linked lectin assay (ELLA) was developed based on these findings. Intriguingly, a correlation between ELLA data and tau levels was observed both in AD and in subjective cognitive impairment.

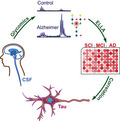

**Abbreviations**
2‐AA: 2‐anthranilic acid
AD: Alzheimer disease
APP: amyloid precursor protein
Aβ: amyloid β‐peptide
CSF: cerebrospinal fluid
def AD: definitive AD
ELLA: enzyme‐linked lectin assay
GlcNAc: glycans carrying bisecting N‐acetylglucosamine
LC: liquid chromatography
MALDI‐TOF: matrix‐assisted laser desorption ionization‐time of flight
MCI: mild cognitive impairment
MS: mass spectrometry
PHA‐E: *Phaseolus vulgaris* Erythroagglutinin
pro AD: probable AD
P‐tau: Phosphorylated tau
SCI: subjective cognitive impairment
T‐tau: total tau

## Introduction

Alzheimer disease (AD) is a devastating progressive neurodegenerative disorder, and the most common form of dementia, with memory impairment as one of the earliest signs. The disease development starts many years before the onset of the first symptoms, and before a clinical diagnosis can be made 1, and still current treatments available are only symptomatic 2, 3. AD pathology is characterized by two pathological hallmarks: extracellular amyloid plaques composed of amyloid β‐peptide (Aβ) 4 and dystrophic neurons filled with neurofibrillary tangles composed of tau protein 5, together with a progressive degeneration and loss of neurons and synapses. Aβ is a 40‐ to 43‐amino‐acid‐long peptide derived from the amyloid precursor protein (APP) after proteolytical processing first by β‐secretase, generating a soluble fragment (sAPPβ) and a membrane‐bound C‐terminal fragment (C99). The latter is then cleaved by γ‐secretase to generate a cytosolic fragment called AICD and the luminal/extracellular Aβ 6. Aβ is known to be a key player in the disease development, since all known forms of hereditary AD with 100% penetrance have mutations in one of three genes, APP, presenilin 1, or presenilin 2, which leads to relatively higher levels of the more neurotoxic variant—Aβ42 7, 8. However, these mutations cause < 1% of all AD cases. The underlying cause of sporadic AD is less clear, but evidence suggests that also in these cases, Aβ aggregation and tau pathology are central in the pathogenesis 9. Tau is a microtubule‐associated protein, which under normal circumstances stabilizes microtubules but in AD becomes hyperphosphorylated and aggregates into paired helical filaments and eventually neurofibrillary tangles 10. Phosphorylated tau (P‐tau) is enriched in tangled neurons, whereas total tau (T‐tau) levels in CSF reflect general neuronal damage 11. Both P‐tau and T‐tau levels are increased in CSF in AD 3.

Increasing evidence reveals connections between AD and protein glycosylation, a protein modification present in at least 50% of all proteins, which plays major roles on biological functions, not least in the brain 12. Glycans can be covalently linked to proteins on Asn residues (N‐glycans) or Ser/Thr residues (O‐glycans) and affect stability and folding properties of proteins and serve as recognition tags in intracellular sorting as well as interactions between proteins and receptors. Several proteins involved in AD pathogenesis, including tau, BACE‐1, and APP, have been found to have altered glycosylation pattern in AD 12, 13, 14, and both N‐ and O‐glycosylation pathways appear to be affected 15, including O‐GlcNAcylation, a protein modification involving addition of one single monosaccharide, N‐acetylglucosamine (GlcNAc), to the protein 16.

**Figure 1:**
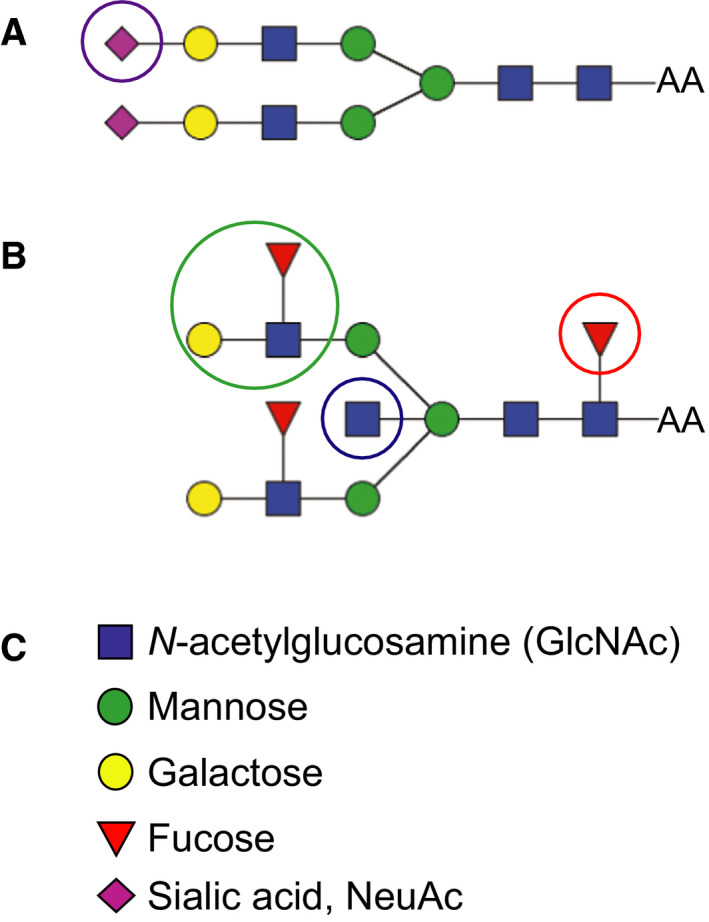
Examples of two different biantennary N‐glycans. (A) Biantennary glycan with sialic acid on both antenna. (B) Biantennary glycan with a core fucose, two Lewis X structures, and a bisecting GlcNAc. (C) The individual monosaccharide units of the N‐glycan structures in A and B. Epitopes of particular interest in this study are marked with circles. Purple circle shows sialic acid (magenta diamond); blue circle shows Lewis X epitope; and red circle shows core fucose.

Intriguingly, tau isolated from AD brain, but not control brain, was found to be N‐glycosylated 17. This was an unexpected finding since tau under normal circumstances is a cytosolic protein and thus not expected to reach the N‐glycosylation machinery in the ER‐Golgi system in the secretory pathway. On the other hand, tau does have three potential N‐glycosylation sites. The aberrant glycosylation of tau in AD was reported to be present both in nonphosphorylated and P‐tau and in paired helical filaments 17, 18. It has been suggested that N‐glycosylation precedes phosphorylation of tau in AD 17, 18, 19 and that the N‐glycans attached to tau affect its phosphorylation and aggregation 19, 20. In contrast to tau, many other proteins involved in AD pathogenesis, including APP, BACE‐1, and nicastrin, are N‐glycosylated under normal circumstances and show alterations in the glycosylation pattern in AD brain 12, 21. Several recent studies have shown that alterations in intracellular transport of APP and BACE1, due to changes in glycosylation, regulate APP processing and Aβ generation 13, 22. For BACE‐1, the levels of the glycan epitope bisecting GlcNAc (see Fig. 1 for explanation) were reported to be altered in AD brain. In line with the altered glycosylation of several proteins in AD, a recent study showed altered expression of enzymes involved in glycosylation pathways in AD brain 23, supporting that there are global alterations in protein glycosylation in AD.

Alterations in protein glycosylation are relatively unexplored in the AD research field although it has high potential to be used both to discover novel biomarkers for AD and for understanding mechanisms behind AD, which may lead to new treatment strategies. We therefore analyzed the N‐glycome in CSF from patients with AD and potential prestages of AD. Cases with subjective cognitive impairment (SCI) report cognitive complaints but do not show significant cognitive deficit upon clinical investigation, while patients with mild cognitive impairment (MCI) have measurable cognitive deficit(s) but are not yet demented. MCI patients are often considered a transition form upon development of AD 24. Our first set of glycomics data obtained by matrix‐assisted laser desorption ionization‐time of flight (MALDI‐TOF) mass spectrometry (MS) revealed dramatic changes in the glycosylation pattern in CSF in AD. We therefore developed a liquid chromatography (LC)−MS approach and a multiwell plate enzyme‐linked lectin assay (ELLA), enabling higher throughput and thus simpler and quicker analysis. Using ELLA to analyze a cohort of 242 patients with SCI, MCI, or AD showed significant differences in the MCI and AD groups compared with the SCI group. Interestingly, a correlation with both T‐tau and P‐tau was observed already at the SCI stage, indicating that this alteration may be a very early event during the progression of the disease.

## Results

### MALDI‐TOF mass spectrometry revealed alterations in relative levels of CSF‐derived N‐glycans in AD and MCI compared to nondemented controls

**Figure 2:**
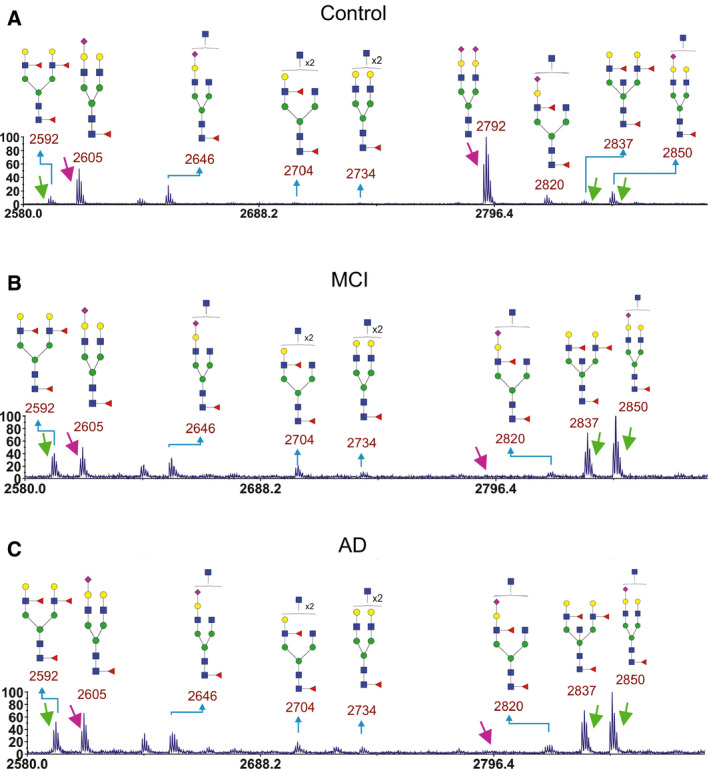
Parts of MALDI‐TOF mass spectra of permethylated CSF‐derived N‐glycans in CSF samples. Results from pooled CSF samples from (A) control (*n* = 31), (B) MCI (*n* = 27), and (C) AD (*n* = 25) are shown. Arrows point at peaks used for ratio calculations of different glycans. Green arrows point at peaks that were increased in MCI and AD compared to control. Magenta arrows point at peaks that were decreased in MCI and AD compared to control.

Our first glycomics profiling of cerebrospinal fluid (CSF) from AD patients was carried out using MALDI‐TOF MS of permethylated N‐glycans after enzymatic removal of the glycans from glycoproteins by PNGase F. Pooled CSF samples from nondemented controls (*n* = 31), MCI (*n* = 25), and AD (*n* = 27) were analyzed. In each sample pool, 30–31 N‐glycans were identified (Fig. S1) and the identified glycans were basically the same in all groups. However, the relative levels of certain glycans differed. Specifically, when examining the spectra in the *m/z* region 2580–2900, there were large differences in MCI and AD compared with the control (Fig. 2), and the peaks at *m/z* 2837 and 2850 (marked with green arrows) were higher relative to the peaks with *m/z* 2605 and 2792 (marked with magenta arrows) in MCI and AD compared to the control (Table 1). The biantennary glycan with sialic acid on both antenna lacking the core fucose observed at *m/z* 2792 in the control was barely detectable in the MCI and AD pools. The glycans at *m/z* 2837 and *m/z* 2850 are consistent with structures having a bisecting GlcNAc based on this set of MALDI‐TOF and MS/MS analyses. The presence of these glycans with bisecting GlcNAc in CSF was later confirmed by electrospray MS/MS (see below). The peak height of the biantennary glycan with a core fucose and Lewis X structures on both antenna observed at *m/z* 2592 was increased relative to the peak at 2605, although this increase was relatively smaller than for the glycans with bisecting (and putative) GlcNAc.

**Table 1:** Ratio of peak heights from MALDI‐TOF MS (*m/z*, permethylated ions, *z* = 1) for selected glycans in CSF. The peak heights were measured from spectra shown in Figs 2 and S1.

	2592/2605	2592/2792	2837/2605	2837/2792	2850/2605	2850/2792
Cntr	0.22	0.12	0.12	0.06	0.35	0.19
MCI	0.83	10	1.5	18	2.0	24
AD	0.80	14	1.1	19	1.5	27

**Figure 3:**
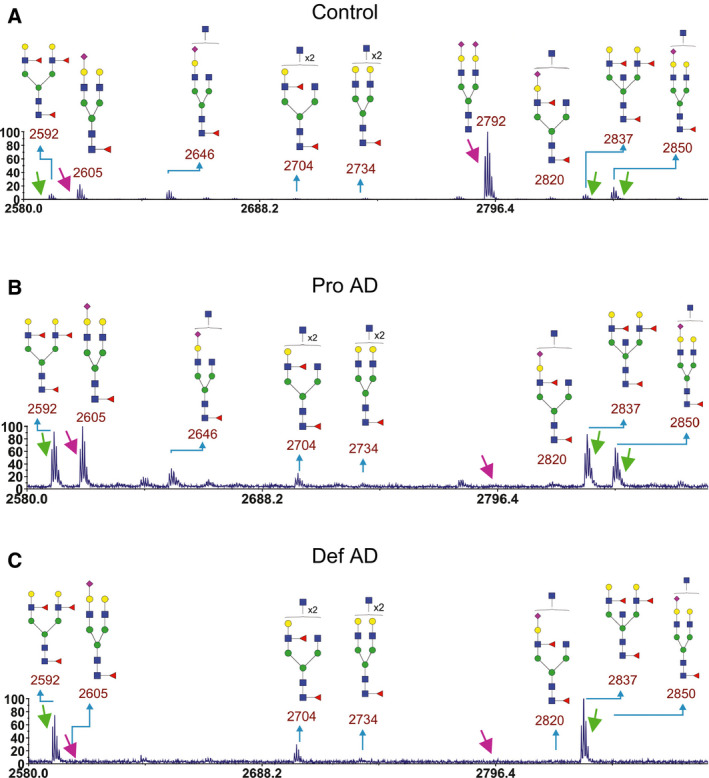
Parts of MALDI‐TOF mass spectra of permethylated N‐glycans from postmortem ventricular fluid. Results from pooled postmortem ventricular fluid from (A) control (*n* = 5), (B) pro AD (*n* = 3), and (C) def AD (*n* = 5) cases are shown. Arrows point at peaks used for ratio calculations of different glycans. Green arrows point at peaks that were increased in MCI and AD compared to control. Magenta arrows point at peaks that were decreased in MCI and AD compared to control.

Diagnosis of AD is complex and typically involves both the clinical assessment and biomarker analyses 25. Still, examination of the postmortem brain can give more detailed information on the type, severity, and distribution of pathology. Therefore, we used also pooled samples of postmortem ventricular fluid from control (*n* = 5), probable AD (pro AD, after postmortem analysis defined as AD, *n* = 3), and definitive AD (def AD, *n* = 5) cases for glycomics analyses. Between 27 and 37 N‐glycans were identified in each sample pool (Fig. S2). The increased number of glycans detected as compared to the CSF samples was accounted for by the pooled control, in which we could detect some triantennary glycans not detected in the other samples. As for CSF samples, we zoomed into the spectra region from *m/z* 2580–2900 (Fig. 3). Similar alterations in glycan ratios between control and AD cases were observed as for CSF samples (Table 2) with the exception that the def AD case had very low levels of the peaks at *m/z* 2605, 2820, and 2850. One possibility is that sialic acid from these glycans was degraded or hydrolyzed during the postmortem time, since sialic acid can be lost if stored for too long a time or under nonoptimal conditions.

**Table 2:** Ratio of peak heights (*m/z* for the permethylated glycans) of selected glycans in postmortem ventricular fluid. The peak heights were measured from spectra shown in Figs 3 and S2.

	2592/2605	2592/2792	2837/2605	2837/2792	2850/2605	2850/2792
Control	0.42	0.09	0.42	0.094	0.83	0.19
Pro AD	0.92	12	0.98	12	0.66	8.3
Def AD	9.5	9.5	12	13	0.75	0.75

Overall, the glycomics analyses of permethylated glycans suggested that there was a clear increase in the levels of certain N‐glycans containing bisecting GlcNAc and Lewis X epitopes.

### Nano‐LC−MS verified enhanced levels of N‐glycans containing bisecting GlcNAc in CSF in AD compared to controls

**Figure 4:**
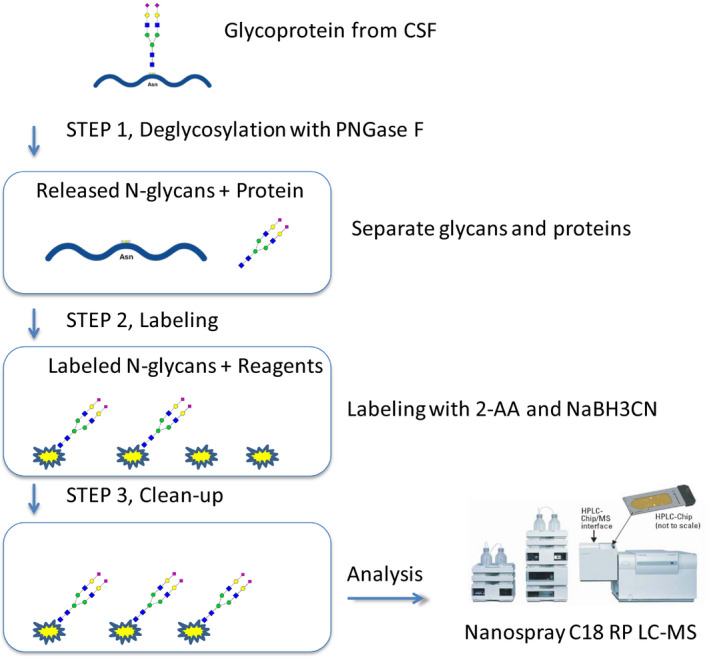
Schematics of LC‐MS/MS method for analysis of 2‐AA‐labeled N‐glycans.

Since MALDI‐TOF MS of permethylated N‐glycans suggested that there are epitope‐specific alterations in the CSF N‐glycome in pooled samples from MCI, pro AD, and AD compared to nondemented controls, we set up additional methods that make it easier to analyze large datasets and obtain data on individual variation. In the first step toward this goal, we used a nano‐LC−MS system employing reversed‐phase chromatography (Fig. 4) for the analysis of individual CSF samples. N‐glycans were labeled with 2‐anthranilic acid (2‐AA) to enhance the detection signal and make the response factor more uniform between different glycans as compared to native glycans. This derivatization also enhances the hydrophobicity of the N‐glycans and thus makes it possible to separate different glycans by reversed‐phase chromatography. Five well‐defined AD cases and four age‐ and sex‐matched control samples were used. A typical LC‐MS base peak chromatogram showing separation between different N‐glycans is shown in Fig. S3. Tandem MS was used for the determination of the N‐glycan structures (Fig. S4).

**Figure 5:**
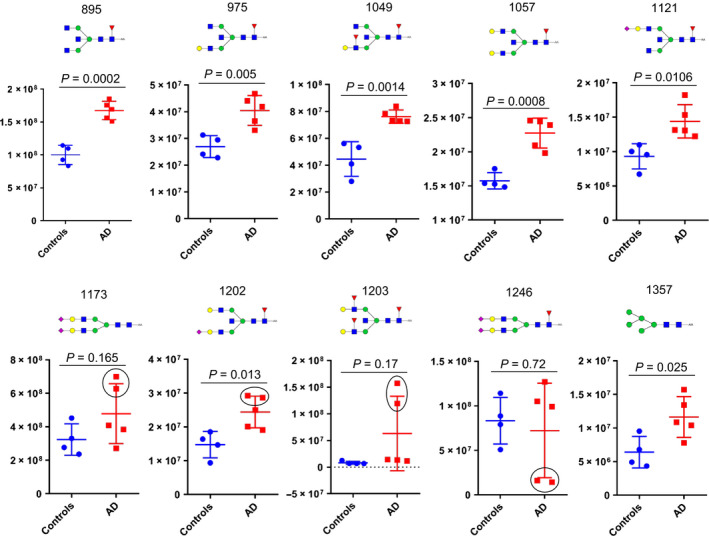
Nanospray LC‐MS/MS of 2‐AA‐labeled N‐glycans. Total area from extracted base peak chromatograms of the ten most abundant N‐glycans was plotted for four control and five AD samples from the Clinical Neurochemistry Laboratory. Lines represent mean values ± SD. The significance between the groups was calculated using two‐tailed unpaired Student’s *t*‐test. The *m/z* value and the cartoon glycan structure are displayed above each graph. Not that two of the AD samples differed from other AD samples for the peaks at *m/z* 1173, 1202.5, 1203, and 1246 (marked with a black circle).

Ten glycans with selected ion traces for *m/z* = 895, 975, 1049, 1057, 1121, 1173, 1202, 1203, 1246, and 1357 were selected for analysis (Fig. 5). The abundance of these peaks was determined by calculating the extracted chromatogram peak area. Seven of these glycans contain a bisecting GlcNAc (*m/z* 895, 975, 1049, 1057, 1121, 1202, and 1203). Six of these glycans were significantly increased in AD compared to control, whereas one (*m/z* 1203), containing Lewis X epitopes on both antenna, was found in remarkably high levels in two of the AD samples, but not the other three AD, suggesting that there is heterogeneity in AD with respect to this glycan.

The glycans lacking bisecting GlcNAc and carrying sialic acid on both antenna (*m/z* 1173 and 1246) were not significantly increased in AD, while the high mannose glycan at *m/z* 1357 was significantly altered in AD but less so than the glycans containing bisecting GlcNAc.

### **Enzyme‐linked lectin assay (ELLA**)** shows increased PHA‐E binding in AD compared to controls**

**Figure 6:**
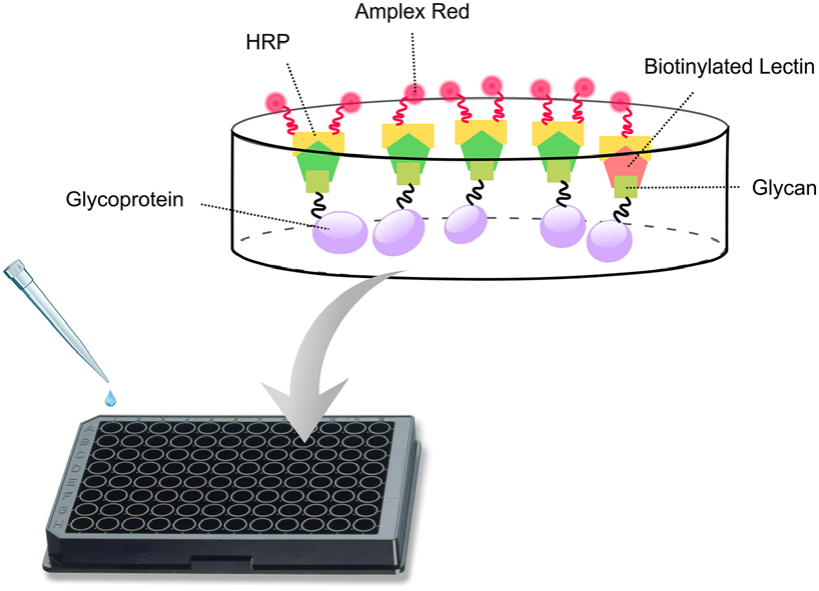
Schematics of ELLA. Diluted CSF samples were incubated in 96‐well high‐absorbing plates. The samples were washed, and biotinylated lectin (PHA‐E) was added. After washing, avidin‐conjugated HRP was added followed by the addition of Amplex red. The fluorescence from the resulting product was measured in a fluorescence reader.

**Figure 7:**
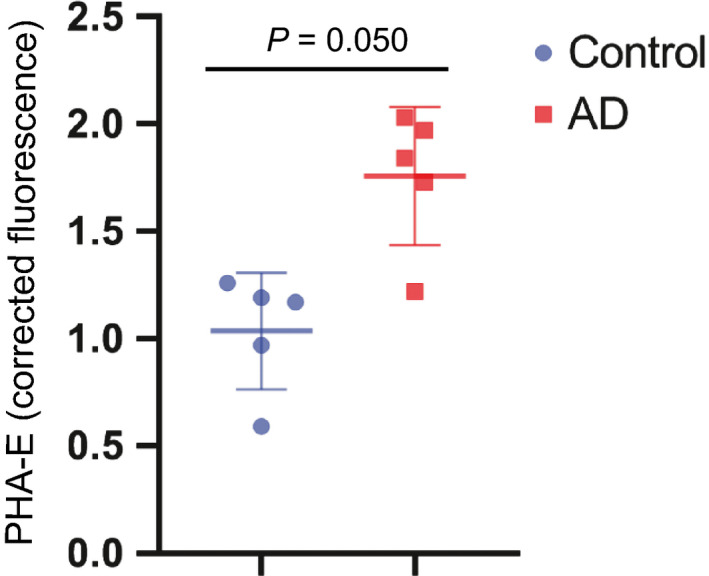
PHA‐E binding to CSF glycoproteins in control and AD cases. Corrected fluorescence signal obtained from ELLA was plotted for replicates of single individuals in control (*n* = 5) and AD (*n* = 5) samples from the Clinical Neurochemistry Laboratory. Lines represent mean values ± SD. Significance between the groups was calculated using two‐tailed unpaired Student’s *t*‐test.

Next, we developed a sensitive assay (ELLA) with high throughput to enable quicker analyses of larger datasets compared with the MS methods. To this end, we used a multiwell plate format and took advantage of the findings from MS experiments showing that the most abundant N‐glycans that were increased in AD compared with the control carried a bisecting GlcNAc. It is based on coating of CSF to high‐absorbing 96‐well plates, detection with biotinylated lectin followed by HRP‐conjugated avidin and Amplex red (Fig. 6). The lectin *Phaseolus vulgaris* Erythroagglutinin (PHA‐E), which is specific for N‐glycans containing bisecting GlcNAc 26, 27, was used. In the development of this assay, we tested different concentrations of all components and incubation times until it was optimized with respect to reproducibility and linearity. The assay is very sensitive and requires as little as 0.05 μL CSF per sample (100 μL of CSF diluted 2000 times per well). We first analyzed the same set of samples as for the nano‐LC/MS analyses (CSF samples from five control and five AD cases from Clinical Neurochemistry Laboratory, University of Gothenburg). Indeed, we saw similar results as in the MS analysis with a significantly increased signal in the AD samples compared to the control (Fig. 7).

**Figure 8:**
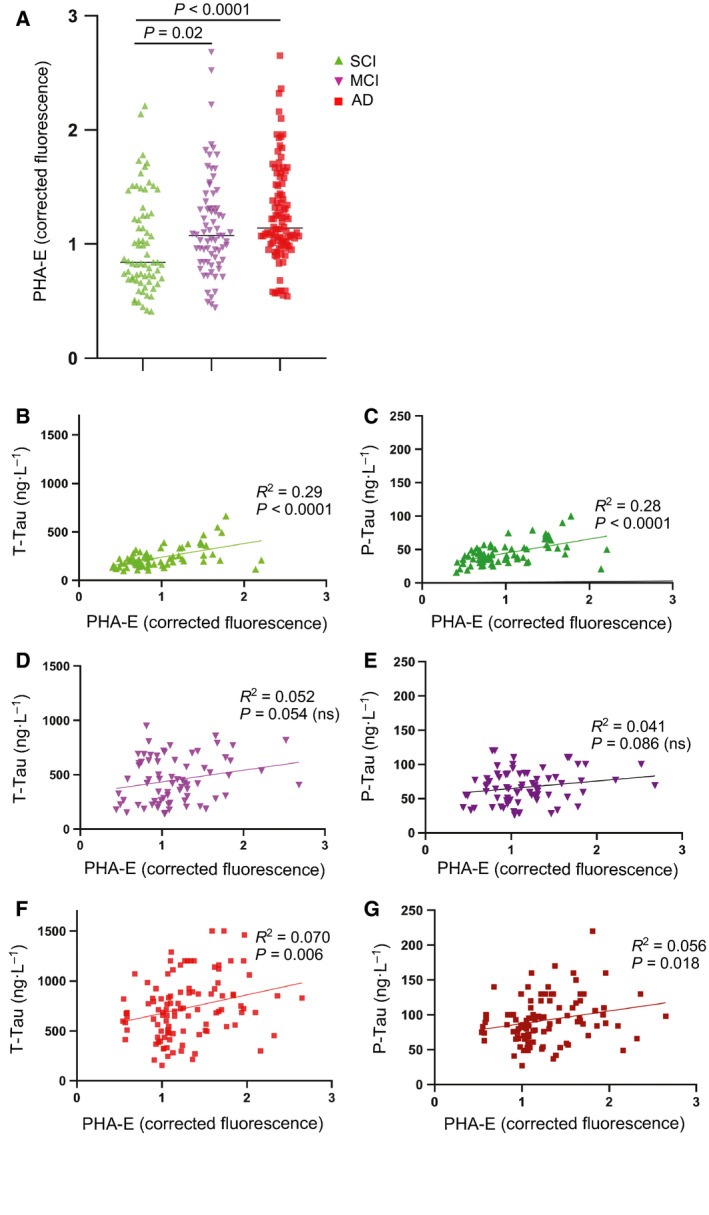
PHA‐E binding to CSF glycoproteins in different patient groups. (A) Corrected fluorescence signal obtained from ELLA was plotted for replicates of single individuals in SCI (*n* = 70), MCI (*n* = 72), and AD (*n* = 100) samples from the GEDOC registry. Significance between the groups was calculated using two‐tailed unpaired Student’s *t*‐test. (B, C) Plots of CSF tau levels vs PHA‐E binding in the SCI group. (D, E) Plots of CSF tau levels vs PHA‐E binding in the MCI group. (F, G) Plots of CSF tau levels vs PHA‐E binding in the AD group. Correlation was determined by calculating *R* ^2^ and *P* values by linear regression in graphpad prism.

Enzyme‐linked lectin assay analysis of a larger set of SCI, MCI, and AD samples from the GEDOC registry (Karolinska, Stockholm) showed significant increases in the MCI and AD groups compared to SCI (Fig. 8A). The distribution was rather heterogeneous in these groups, which may indicate that within the SCI, MCI, and AD groups, there are mixed populations including that some of the SCI and MCI cases will develop AD and also the AD group is heterogeneous.

**Figure 9:**
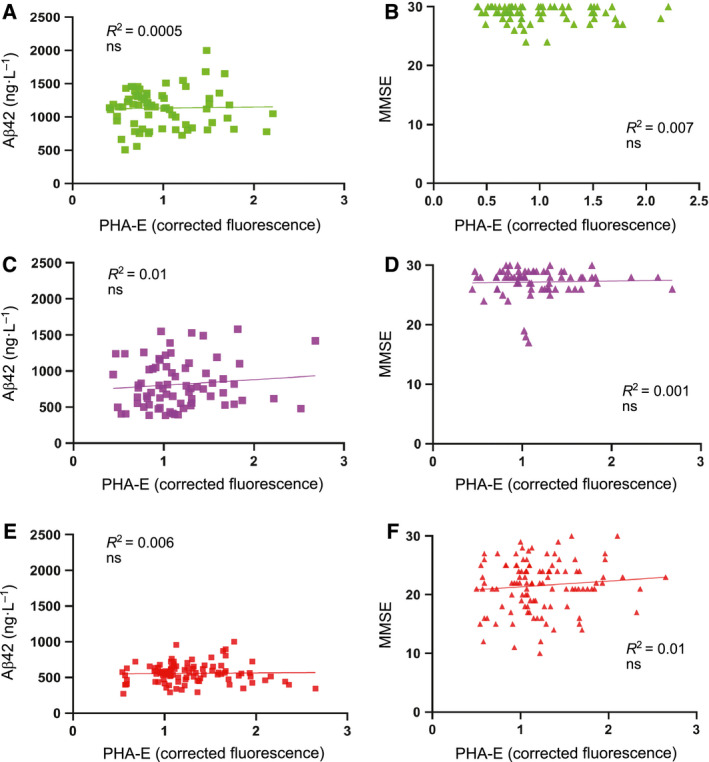
Correlation of PHA‐E binding to CSF glycoproteins with MMSE and CSF levels of Aβ42. The samples are the same as in Fig. 6. (A, B) Plots of Aβ42 (A) and MMSE (B) vs PHA‐E binding in the SCI group. (C, D) Plots of Aβ42 (C) and MMSE (D) vs PHA‐E binding in the MCI group. (E, F) Plots of Aβ42 (E) and MMSE (F) vs PHA‐E binding in the AD group. Correlation was determined by calculating *R* ^2^, and significance was determined by calculating *P* values by linear regression in graphpad prism.

For the CSF samples used in this study, data on standard biomarkers were already available, which made it possible to compare ELLA data with other biomarkers. Interestingly, correlation studies showed a significant correlation with CSF T‐tau and P‐tau, particularly in the SCI group (Fig. 8B,C). A similar trend was observed in MCI (Fig. 8D,E) and AD (Fig 8F,G), although it was not significant in MCI and less significant in AD than in SCI. In contrast, no significant correlation with CSF Aβ42 or MMSE was observed in either SCI, MCI, or AD (Fig. 9).

## Discussion

In the search for mechanisms involved in AD pathogenesis and to identify novel biomarkers for AD, we performed glycomics of CSF samples from healthy controls and patients with SCI, MCI, and AD. Indeed, we found alterations in the glycosylation pattern in MCI and AD compared with the controls using two different MS methods. A relative increase in glycans containing bisecting GlcNAc (Fig. 1B) was found with both MS methods used. Both methods also suggested a relative increase in the levels of glycans containing Lewis X (Fig. 1B), although the nano‐LC/MS method suggested heterogeneity in AD with respect to the levels of Lewis X‐containing glycans. In contrast, the observation that a biantennary glycan with sialic acids on both antenna and lacking core fucose (Fig. 1A) was highly abundant in the control group but barely detectable in MCI and AD with MALDI‐TOF (*m/z* 2792 in Figs 2 and 3) was not reproducible with nano‐LC−MS. When individual CSF samples were analyzed with our LC‐MS method, we observed a high variation in the levels of this glycan (*m/z* 1173 in Fig. 5) between different individuals. Interestingly, a high variability of the levels of this glycan in CSF samples from MCI and AD cases was previously reported 28. In the same study, it was found that patients in the MCI group that had low levels of this glycan, combined with high levels of three glycans containing bisecting GlcNAc, developed AD, whereas the MCI patients with glycosylation pattern more similar to the controls did not 28. Clearly, it would be interesting to analyze such correlations with larger patient groups in future investigations to clarify whether altered protein glycosylation represents a risk factor for developing AD.

Since the levels of glycans containing bisecting GlcNAc were elevated in CSF from AD patients with two different methods and sample materials, we decided to continue our study in larger patient groups. We therefore optimized the simple, highly sensitive, and robust multiwell plate assay, ELLA, for this purpose. The lectin used in this assay, PHA‐E, has been shown in several studies to have high specificity for bisecting GlcNAc present in N‐glycan structures 26, 27, 29. Indeed, using this method on a cohort with CSF samples from 242 patients, despite a substantial heterogeneity in all three groups (SCI, MCI, and AD), we found significant increases in both the MCI and AD groups compared with the SCI group.

The finding that bisecting GlcNAc‐containing glycans, measured by ELLA, correlated with CSF T‐tau and P‐tau levels but not with Aβ42 or cognition, as evaluated by MMSE, and that the correlation was particularly pronounced in SCI, is intriguing. It also raises several questions and one question is whether we are detecting bisecting GlcNAc attached to tau protein or whether tau is rather affected by other proteins modified with bisecting GlcNAc. The previous finding that tau isolated from AD brain contains N‐glycans with bisecting GlcNAc 30 supports the possibility that this form of Tau in CSF contributes to the correlation. The presence of N‐glycans on tau in AD brain is unexpected since tau is a cytosolic protein and cytosolic proteins are normally not N‐glycosylated. However, several studies, including lectin blotting and monosaccharide composition analysis of tau isolated from AD brain, double immunostaining of AD brain sections, and deglycosylation with N‐glycan‐specific glycosidases, support this notion 17, 19, 30, 31, 32. Moreover, a recent study showed that tau becomes N‐glycosylated and, as a result, becomes more aggregation‐prone, when forced through the secretory pathway in a SH‐SY5Y cell‐based model 20.

It is well known that in CSF from AD patients, tau levels are increased, while Aβ42 levels are decreased, and a combination of T‐tau, P‐tau, and Aβ42 is commonly used as biomarker for diagnosis of AD 3. However, it is not clear why this is the case, since both tau and Aβ42 aggregate in AD brain. One potential explanation could be that tau passes through the secretory pathway in AD and becomes glycosylated, in contrast to controls where it remains cytosolic. Such a scenario could explain why glycosylated tau becomes secreted to a higher extent in AD than nonglycosylated tau in healthy brain. In favor of this hypothesis, the study with a SH‐SY5Y cell model expressing tau fused to a signal peptide targeting tau to the secretory pathway showed that tau became N‐glycosylated and secreted when forced through the secretory pathway 20. Other studies have shown that Aβ induces secretion of tau fragments 33 and much of the tau in CSF is in the form of fragments 34. Another possibility could thus be that increased tau in CSF is derived from degenerating neurons. However, if the latter was the case, it would seem reasonable that the correlation was higher in AD with massive neurodegeneration than in SCI. Interestingly, our study shows the opposite trend, that is, higher correlation between bisecting GlcNAc and tau in SCI than in AD, favoring the former hypothesis. Thus, it will be interesting in future investigations to determine whether the increased levels of Aβ42 in AD brain, which starts several years before the development of clinical symptoms 35, induce an alteration in the N‐glycosylation pathways.

Previous studies have shown interesting connections between BACE1 and bisecting GlcNAc, since BACE1 modification with bisecting GlcNAc is increased in AD brain 13. The presence of bisecting GlcNAc on BACE‐1 was suggested to target the enzyme to the early endosomal compartment where it encounters and processes APP thus generating Aβ, whereas BACE1 lacking bisecting GlcNAc is targeted to the late endosomal/lysosomal compartment where it is degraded. Moreover, MGAT3 deficiency (i.e., loss of the enzyme that adds a bisecting GlcNAc to N‐glycans) reduced Aβ plaque load in transgenic mice expressing hAPP carrying the Swedish mutation. Those previous data together with the present findings suggest that the reported enhanced activity of MGAT3 in AD may affect several pathological pathways in AD.

## Conclusions

The alterations in the N‐glycan profile in CSF, with consistent increased levels of glycans containing bisecting GlcNAc, Lewis X epitopes, and, less consistently, decreased levels if biantennary glycans with sialic acid on both antenna, occur at SCI and MCI stages, which may represent early phases during AD development. These findings could thus be important both for developing early biomarkers for AD and for understanding early stages of AD development, which may be used for designing novel treatment strategies.

## Materials and methods

### Subjects

MALDI‐TOF MS of permethylated N‐glycans was performed on pooled CSF samples from 31 control patients, 25 MCI patients, and 27 AD patients, in a total volume of 50–100 µL in each pool, from Huddinge hospital and the GEDOC registry. MALDI‐TOF MS was also performed on pooled postmortem ventricular fluid samples from five control cases, three pro AD cases (pro AD, after postmortem analysis defined as AD), and five def AD cases. Nano‐LC‐tandem MS was performed on de‐identified left‐over CSF samples from four control and five AD patients from the Clinical Neurochemistry Laboratory in Mölndal. The patients sought medical advice because of early cognitive impairment or other mild psychiatric symptoms, for example, stress‐related and were designated as normal or AD according to criteria described previously 36, 37. Mean concentrations of the AD CSF biomarkers in the AD and non‐AD groups were 1155 ± 307 ng·L^−1^ and 264 ± 60 ng·L^−1^ for CSF T‐tau, 120 ± 15 ng·L^−1^ and 45 ± 6 ng·L^−1^ for CSF P‐tau, and 386 ± 95 ng·L^−1^ and 795 ± 199 ng·L^−1^ for CSF Aβ42. ELLA was performed on 242 patients classified as SCI, MCI, or AD, from the GEDOC registry. This study population was diagnosed according to criteria described previously 38. The cases were not diagnosed with any other neurodegenerative diseases but we cannot exclude that some of them suffer from other diagnosis. CSF was usually taken at the time of diagnosis. CSF biomarkers for this study group are plotted in Figs 8 and 9. The three different datasets used in this study are further summarized in Table 3. The experiments were undertaken with the understanding and written consent of each subject. The study methodologies conformed to the standards set by the Declaration of Helsinki. The study methodologies were approved by the local ethics committees.

**Table 3:** CSF and postmortem ventricular fluid samples used in this study. NA: not applicable.

	Number of samples	Mean age at sample collection	Males/females	Postmortem time
Postmortem ventricular fluid				
Cntr	5	83	1/4	22 ± 12
Pro AD	3	83	0/3	21 ± 8
Def AD	5	83	0/5	23 ± 13
CSF samples from the Clinical Neurochemistry Laboratory, Mölndal				
Cntr	5	71	2/3	NA
AD	5	75	3/2	NA
CSF samples from GEDOC registry, Karolinska hospital, Huddinge				
SCI	70	57 (onset)	36/34	NA
MCI	72	65	36/36	NA
AD	100	71	48/52	NA

### Preparation and permethylation of N‐glycans for MALDI‐TOF mass spectrometry

The samples were reduced by dithiothreitol, carboxymethylated by iodoacetic acid, and dialyzed and digested by trypsin as described previously 39. N‐glycans were liberated from the resulting peptides by PNGase F (Roche Applied Science, Penzberg, Germany) and separated from the peptides by C18 Sep‐Pak chromatography (Waters Corporation, Milford, MA, USA). The liberated glycans were permethylated with the sodium hydroxide procedure as described earlier 40.

### MALDI‐TOF mass spectrometry

MALDI‐TOF data were acquired on a 4800 MALDI‐TOF/TOF mass spectrometer (PerSeptive Biosystems, Framingham, MA, USA) in the reflectron positive mode with delayed extraction. Permethylated samples were dissolved in 10 μL of methanol, and 1 μL of dissolved sample was premixed with 1 μL of matrix (10 mg·mL^−1^ 3,4‐diaminobenzophenone in 75% (v/v) aqueous acetonitrile for 4800 experiments). MS/MS experiments were performed on the 4800 MALDI‐TOF/TOF mass spectrometer with a collision energy of 1 kV, and argon was used as collision gas. The 4700 Calibration Standard Kit, calmix (Applied Biosystems Sciex, Framingham, MA, USA), was used as the external calibrant for the MS mode, and [Glu1] fibrinopeptide B human (Sigma Aldrich, St. Louis, MO, USA) was used as an external calibrant for the MS/MS mode. The MS and MS/MS data were processed using data explorer 4.9 Software (Applied Biosystems). The processed spectra were subjected to manual assignment and annotation with the aid of a glycobioinformatics tool, GlycoWorkbench 41.

### Preparation of N‐glycans for nano‐LC/MS

The preparation and labeling protocol used for the analysis of N‐glycans by nano‐LC/MS is shown in Fig. 4 **.** N‐glycans were released from CSF glycoproteins by denaturation of 25 µL CSF in 0.1% RapiGest for 15 min at 37 °C followed by addition of five units (NEB units) PNGase F and incubation at 37 °C overnight. The following day, 2–3 additional units of PNGaseF were added followed by incubation for 3 h. The liberated N‐glycans were separated from proteins by 100 µL C18 OMIX tips (Agilent Technologies, Santa Clara, CA, USA). Peptides and proteins bind to the C18 solid phase and can be separated from the free N‐glycans. The tips were first equilibrated by pipetting two times with 60% acetonitrile and 0.2% formic acid in H_2_O and two times with 0.2% formic acid in H_2_O. 50 µL CSF sample was pipetted up and down five to ten times. Nonbound N‐glycans in the flow‐through and in two consecutive washing steps with 0.2% formic acid in H_2_O were collected, pooled, and lyophilized in a Speed‐Vac.

### Labeling of N‐glycans with anthranilic acid

To enhance the detection signal of the N‐glycans, reduce the heterogeneity of signal strength from different glycans, and enable separation by C18 chromatography, we derivatized the glycans with 2‐AA prior to analysis by nano‐LC–MS (Fig. 4). The labeling of N‐glycans with 2‐AA was performed by reductive amination using a protocol adapted from Sigma‐Aldrich. A labeling solution containing 60 mg·mL^−1^ 2‐AA and 6 0 mg·mL^−1^ sodium cyanoborohydride in acetic acid : DMSO (30 : 70) was heated briefly in a 65 °C heating block followed by addition of H_2_O to a final concentration of 10%. Five microliters of this labeling solution was added to the lyophilized N‐glycans and incubated at 65 °C for 3 h. The reaction was stopped by the addition of 95 µL H_2_O, and the labeled N‐glycans were immediately purified and desalted by size exclusion chromatography using PD minitrap G‐10 columns (GE healthcare, Chicago, Ill, USA). The columns were equilibrated with 10 mL H_2_O prior to the application of sample in a volume of 100 µL. After washing with 850 µL H_2_O, labeled N‐glycans were eluted in 250 µL H_2_O. This fraction was stored at −20 °C until the sample was analyzed by nano‐LC/MS.

### Nano‐LC/MS electrospray

2‐AA‐labeled N‐glycans were analyzed with an Agilent 6300 MS LC‐MS system consisting of a 1200 LC system connected to an Agilent chip cube with integrated ion source serving as the interface to a an Agilent 6300 ion trap. Briefly, samples (6 µL per injection, corresponding to 0.8 µL CSF starting material) were separated on a 150 mm Zorbax‐300SB‐C18 chip column with integrated trap column (Agilent, G 4240‐62010) with increasing concentration of acetonitrile (0.2% formic acid in H_2_O in pump system A and 0.2% formic acid in acetonitrile in pump system B). A flow rate of 3 µL·min^−1^ was used, and the gradient was performed in a stepwise manner: 0–1 min, 1% buffer B; 1–2 min, 1–4.5% buffer B; 2–34 min, 4.5–8.5% buffer B; 35–38 min, 95% buffer B; 40–50 min, 1% buffer B.

### LC‐MS Data analysis

Identification of glycan structures was based on MS/MS interpretation. Tandem MS fragments annotation was done manually but facilitated with the Java‐based cross‐platform software GlycoWorkbench 41 that allows *in silico* fragmentation of glycans and drawing of glycan cartoons. LC‐MS figures were drawn in Adobe Illustrator (Adobe Systems, San Jose, CA, USA). The abundance of selected peaks was calculated by manual integration of peak area from extracted ion chromatograms. The peaks were compared between AD and controls in box‐plots. Integration of extracted ion peak areas was done in Agilent Data Analysis and MS Excel. Box‐plots were done in graphpad prism (GraphPad Software Inc, La Jolla, CA, USA).

### Enzyme‐linked lectin assay (ELLA)

A multiwell plate assay was designed (Fig. 6) for analyses of glycan epitopes found to be altered in AD by the MS analysis of CSF‐derived N‐glycans. The concentrations of all components involved as well as incubation times were optimized by titrations in order to obtain the most robust and reliable result. CSF samples were diluted 2000 times in PBS buffer and immobilized in multiwell plates by addition of 100 μL of each diluted CSF sample to Nunc MaxiSorp black flat‐bottom 96‐well plates (Thermo Fisher Scientific, Chicago, Ill, USA) followed by incubation at 4 °C over night. After washing five times with PBST (PBS containing 0.1% Tween‐20, pH 7.4), 110 μL of biotinylated lectin (1 μg·mL^−1^ in PBST) was added to each well and incubated for 2 h at room temperature with shaking. Biotinylated PHA‐E lectin (Vector Laboratories, Burlingame, CA, USA) was used for detecting N‐glycans containing bisecting N‐acetylglucosamine (GlcNAc). This lectin has high specificity to N‐glycans containing bisecting GlcNAc 26, 27. The plates were then washed five times with PBST, followed by the addition of 110 μL of 50 ng·mL^−1^ horseradish peroxidase‐conjugated streptavidin to each well and incubated for 1 h at room temperature with shaking. The plates were washed five times with PBST, followed by addition of 110 µL of 50 µm of Amplex Ultra Red (Invitrogen, Waltham, MA, USA) to each well and incubation for 2 h at room temperature in the darkness. The resulting fluorescence signal was measured at 544 nm excitation and 590 nm emission using a FLUOstar galaxy plate reader (BMG Labtech, Ortenberg, Germany). All CSF samples were analyzed with replicates. In order to further minimize the experimental error, a pool of several CSF samples was used as a standard to normalize the signal of each CSF sample.

## Conflict of interest

KB has served as a consultant or at advisory boards for Axon, Biogen, CogRx, Lilly, MagQu, Novartis, and Roche Diagnostics, and is a cofounder of Brain Biomarker Solutions in Gothenburg AB, a GU Venture‐based platform company at the University of Gothenburg, all unrelated to the work presented in this paper.

## Author contributions

SSW, SG, PS, QC, SMH, AD, and LOT planned experiments; SSW, SG, PS, and QC performed experiments; SSW, SG, PS, QC, SMH, KB, BW, AD, and LOT analyzed and/or interpreted data; KB contributed samples; and SSW, SG, PS, and LOT wrote the manuscript.

## Supporting information


**Fig. S1** **.** MALDI‐TOF mass spectra of permethylated CSF‐derived N‐glycans.
**Fig. S2** **.** MALDI‐TOF mass spectra of permethylated N‐glycans from postmortem ventricular fluid.
**Fig. S3** **.** Extracted LC‐MS base‐peak chromatogram of selected glycans for a typical control sample.
**Fig. S4** **.** MS/MS spectrum of the molecular ion* m/z *1202.92H+ representing a trifucosylated bisecting GlcNAc structure.
