# Supplementary material for: Glycan biomarkers for Alzheimer disease correlate with T‐tau and P‐tau in cerebrospinal fluid in subjective cognitive impairment
Source: FEBS J. 2020 Jan 14;287(15):3221–34. doi: 10.1111/febs.15197 (PMC7496940; doi:10.1111/febs.15197)
Supplement: Supplementary file 1 — Fig. S1 . MALDI‐TOF mass spectra of permethylated CSF‐derived N‐glycans. Fig. S2 . MALDI‐TOF mass spectra of permethylated N‐glycans from postmortem ventricular fluid. Fig. S3 . Extracted LC‐MS base‐peak chromatogram of selected glycans for a typical control sample. Fig. S4 . MS/MS spectrum of the molecular ion m/z 1202.92H+ representing a trifucosylated bisecting GlcNAc structure. [file FEBS-287-3221-s001.zip › febs15197-sup-0001-FigS1-S4.pdf]

FEBS PRESS
science publishing by scientists
WILEY
The FEBS Journal
www.febsjournal.org

Glycan biomarkers for Alzheimer disease correlate with T-tau and P-tau in cerebrospinal fluid in subjective cognitive impairment

Sophia Schedin-Weiss, Stefan Gaunitz, Ping Sui, Qiushi Chen, Stuart M. Haslam, Kaj Blennow, Bengt Winblad, Anne Dell and Lars O. Tjernberg

DOI: 10.1111/febs.15197

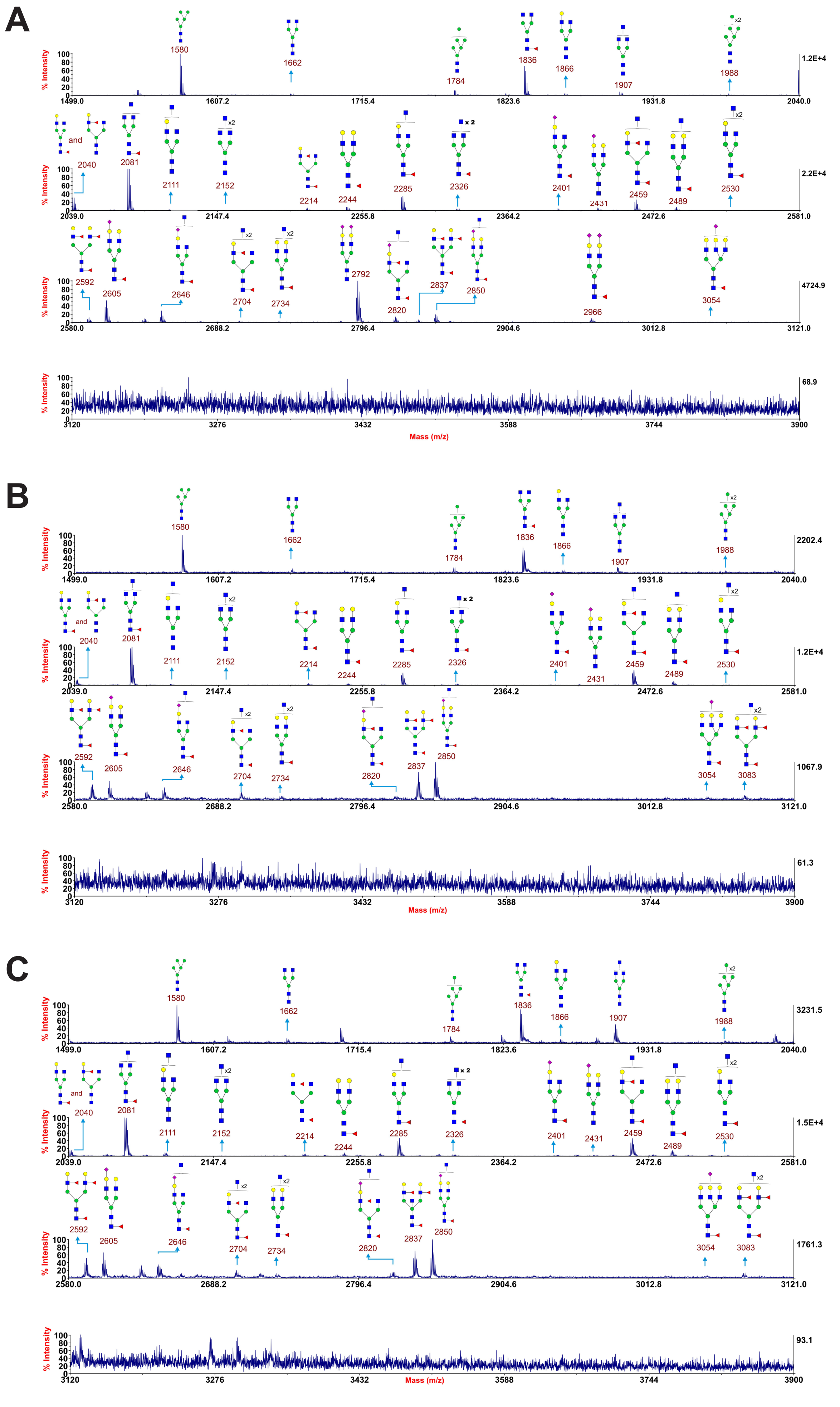

Figure S1. MALDI-TOF mass spectra of permethylated CSF-derived N-glycans. Pooled CSF samples from (**A**) control (n=31), (**B**) MCI (n=27) and (**C**) AD (n=25) were used.

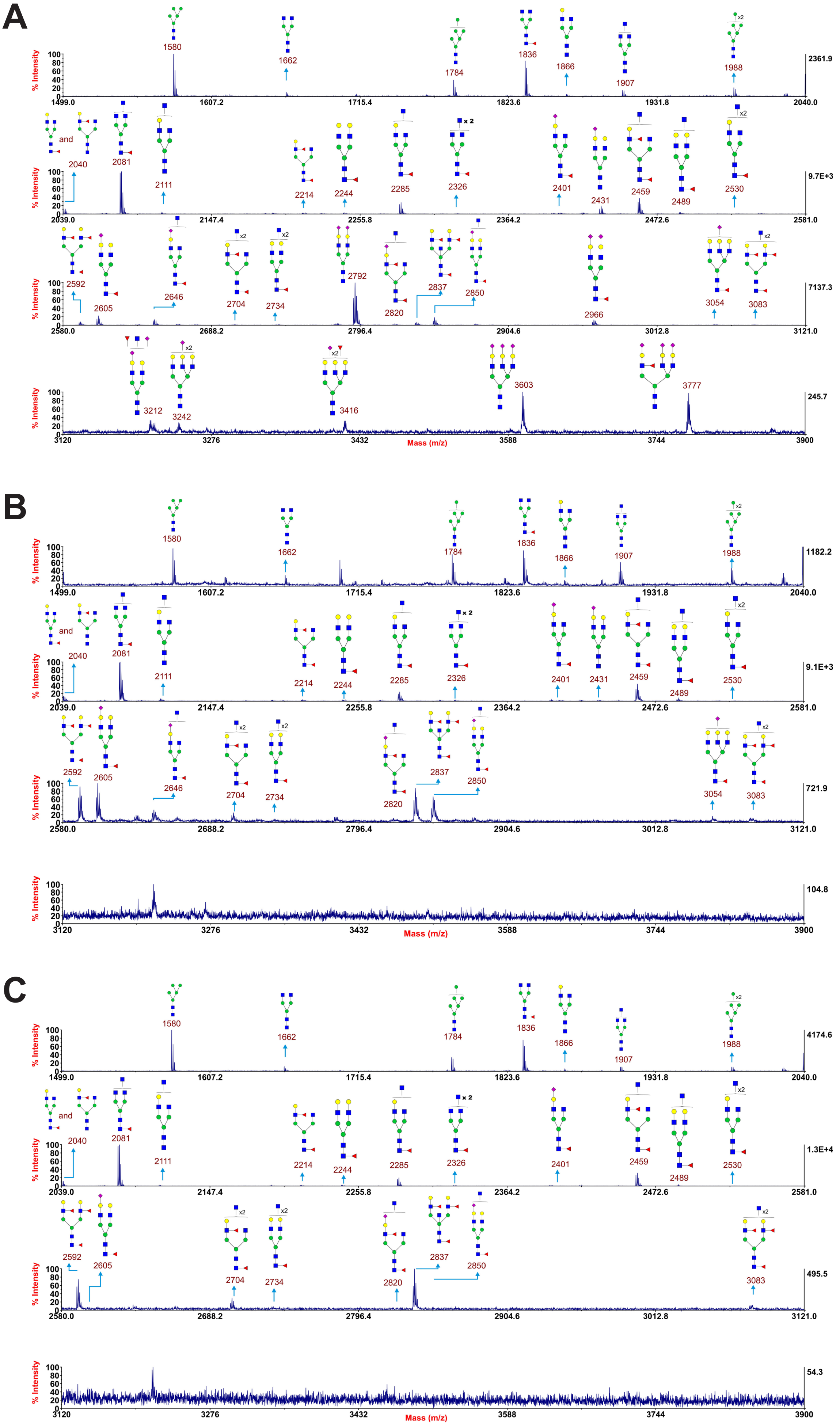

Figure S2. MALDI-TOF mass spectra of permethylated N-glycans from postmortem ventricular fluid. Pooled samples from (**A)** control (n=5), (**B)** probable AD (pro AD, n=3) and **(C)** definitive AD (def AD, n=5) cases were used.

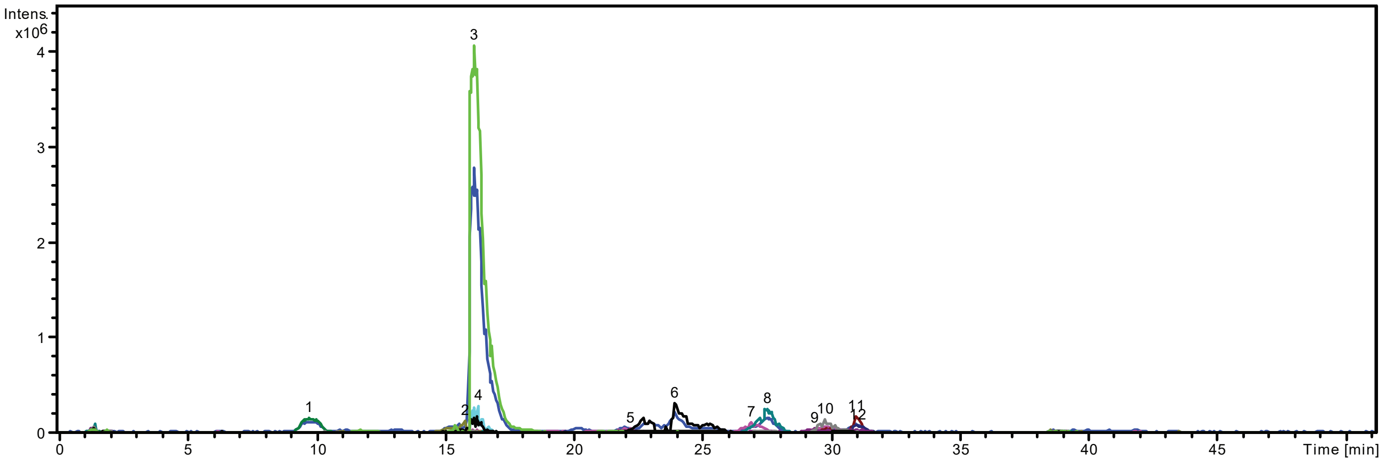

Figure S3. Extracted LC-MS base-peak chromatogram of selected glycans for a typical control sample.

m/z peaks are shown for:

1. 1087.5 (z=2)
2. 1357.1 (z=1)
3. 783.2 (z=2)
4. 1173.4 (z=2)
5. 1202.9 (z=2)
6. 1246.5 (z=2)
7. 1202.0 (z=2)
8. 1048.8 (z=2)
9. 1121.0 (z=2)
10. 1057.2 (z=2)
11. 895.0 (z=2)
12. 975.5 (z=2)

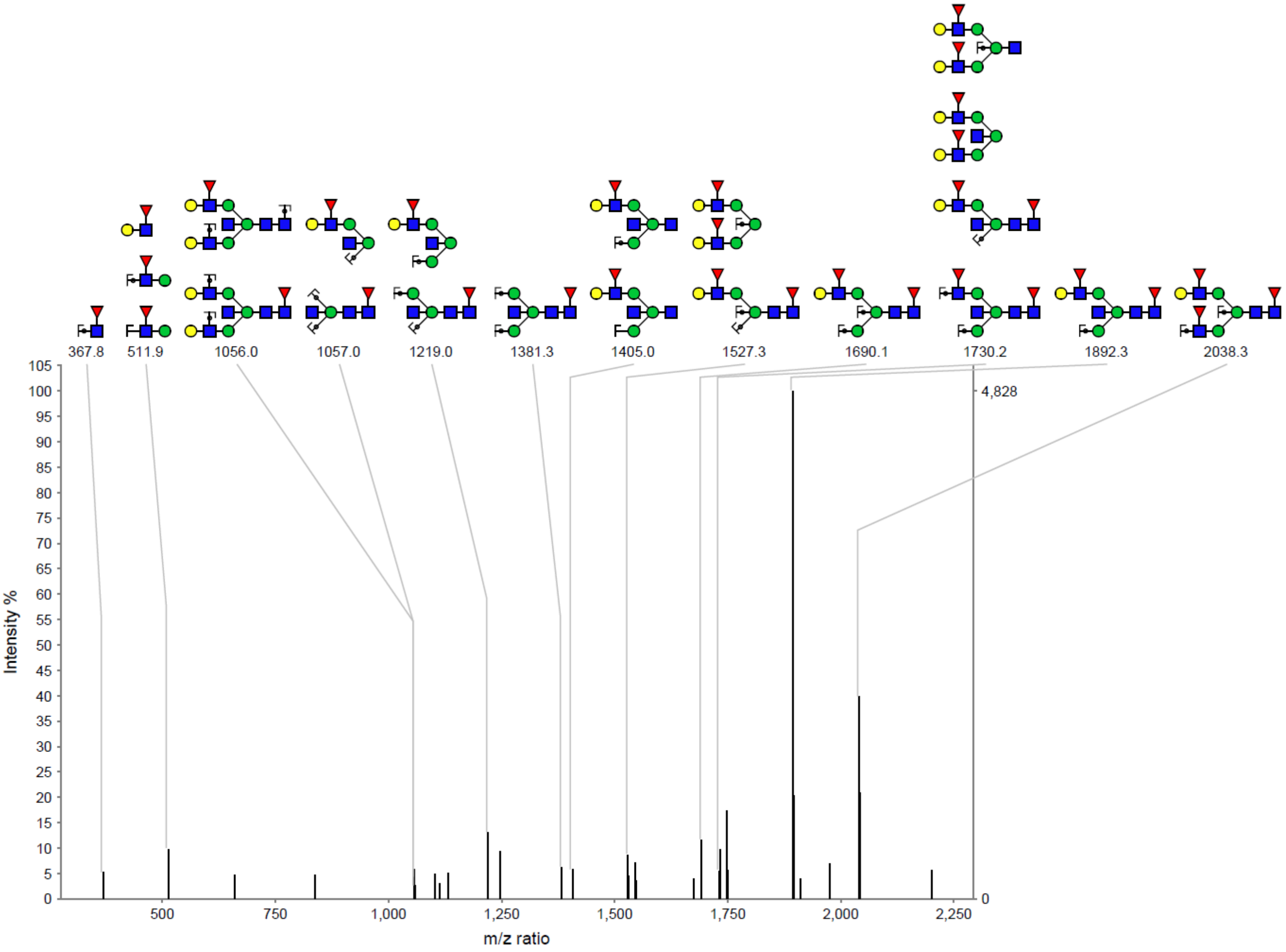

Figure S4. MS/MS spectrum of the molecular ion m/z 1202.9^{2H+} representing a trifucosylated bisecting GlcNAc structure.
The major fragment in the spectrum at m/z 1892.3^{H+} represents loss of the antenna observed at m/z 511.9^{H+} (Hex-(dHex)HexNAc-). The second largest fragment ion, m/z 2038.3^{H+}, matches the loss of a terminal Hex and a terminal HexNAc, most likely representing terminal Gal and bisecting GlcNAc. Bisecting GlcNAc is supported by several diagnostic fragment ions. The fragment at m/z 1219.0 indicates loss of the fucosylated arm (m/z 511.9) and the other arm together with a core mannose (Hex-(dHex)HexNAc-Hex-). The fragment observed at m/z 1057.0 represents loss of both antennary arms including the mannoses, leaving the fucosylated core with one core mannose and the bisecting GlcNAc.
